# Nutritional Recovery and Its Predictors among Adult HIV Patients on Therapeutic Feeding Program at Finote-Selam General Hospital, Northwest Ethiopia: A Retrospective Cohort Study

**DOI:** 10.1155/2020/8861261

**Published:** 2020-12-28

**Authors:** Gedefaw Diress, Nurilign Abebe Moges

**Affiliations:** ^1^Public Health Department, Health Sciences College, Woldia University, Woldia, Ethiopia; ^2^Public Health Department, Health Sciences College, Debre Markos University, Debre Markos, Ethiopia

## Abstract

**Background:**

Undernutrition is a major public health problem in HIV patients in sub-Saharan Africa. To address the problem of malnutrition, the Ethiopian Ministry of Health implemented a therapeutic feeding program, which is the provision of nutritional treatment, care, and support for undernourished individuals. However, little is known about the outcome of a therapeutic feeding program. Therefore, this study aimed to assess nutritional recovery and its predictors among undernourished HIV patients enrolled in a therapeutic feeding program in Northwest Ethiopia.

**Methods:**

An institutional-based retrospective cohort study was conducted among 376 randomly selected adult undernourished HIV patients enrolled in the therapeutic feeding program from July 2010 to January 2017 at Finote-Selam General Hospital. Data were collected by reviewing patients' charts, follow-up cards, and undernutrition treatment registration books using a pretested structured checklist. The main outcome variable was nutritional recovery, defined based on body mass index. Bivariable and multivariable log-binomial regression models were used to identify the predictors of nutritional recovery.

**Result:**

From total undernourished HIV patients enrolled in the therapeutic feeding program, 61.2% were recovered with a median recovery time of 12 weeks (IQR 9–17 weeks) for moderate acute malnutrition and 25 weeks (IQR 22–31 weeks) for severe acute malnutrition. Rural residence (adjusted risk ratio (ARR) = 0.53, 95% CI: 0.27–0.85), no formal education (ARR = 0.24, 95% CI: 0.13–0.54), poor ART adherence level (ARR = 0.14, 95% CI; 0.08–0.32), and WHO clinical stage III or IV (ARR = 0.38, 95% CI; 0.17–0.59) decrease the probability of nutritional recovery.

**Conclusion:**

Nutritional supplementation plays a critical role in the nutritional care and treatment of malnourished patients. Healthcare providers should give more attention to persons with poor adherence levels, advanced WHO clinical stage, rural residence, and low educational status. Future prospective follow-up studies should be performed to assess important variables such as family income, food sharing at the household level, and distance to health institutions.

## 1. Introduction

In mid-2017, 20.9 million people living with HIV were taking antiretroviral therapy (ART) globally [1]. The World Health Organization (WHO) identifies the existence of HIV infection associated with caloric or protein malnutrition and micronutrient deficits. HIV compromises nutritional status, and malnutrition increases susceptibility to opportunistic infections [2, 3]. Sub-Saharan Africa, due to high levels of food insecurity, has the highest proportion of undernourished people in the world [4, 5], along with the highest number of people living with HIV. Nearly 67% of people living with HIV/AIDS were found in this region [4]. In Ethiopia, 1.5% of adult people aged 15–49 were infected with HIV [6].

There is a significant association between HIV infection and undernutrition. People with HIV are exposed to nutritional deficiencies, and their nutritional status is a strong prognostic factor for the progress of the disease, survival, and functioning levels in the course of the disease. Malnutrition significantly increases the mortality risk for HIV-infected individuals regardless of treatment status. Thus, nutritional support is often identified as one of the most immediate and critical needs for people living with HIV [7, 8].

Nutrition counseling and interventions can slow or reverse the process that leads to weight loss and wasting in people living with HIV/AIDS. In addition, the provision of nutritional treatment, care, and support for those undernourished PLHIVs is important to prevent morbidity and mortality [9]. Several organizations such as WHO and United Nations International Children Funds (UNICEF) recommend fortified supplements for managing undernourished cases as an emergency community management to improve nutritional status [10].

In Ethiopia, the prevalence of undernutrition in HIV-infected people was significantly high (12–25%) [9, 11, 12]. In response to this, the Ethiopian Nutritional Therapeutic Feeding Program (TFP) has been implemented by Save the Children, USAID/Ethiopia, and the Ethiopian Ministry of Health since 2010, which provides therapeutic food along with nutritional assessment and counseling to malnourished HIV-infected individuals. In this program, adult HIV-infected people with moderate acute malnutrition (body mass index (BMI) = 16–18.49 kg/m^2^) are provided with two sachets of ready-to-use therapeutic food (RUTF) daily until recovery from malnutrition or for a maximum of three months. Those with severe acute malnutrition (BMI < 16 kg/m^2^) are provided with four sachets daily until recovery or for a maximum of six months [13].

Despite the nutritional therapy program being implemented in all hospitals in Ethiopia, evidence on the outcome of nutritional therapeutic feeding interventions among adult HIV patients is still limited. The Ethiopian Ministry of Health and other stakeholders need evidence on the progress of the nutritional intervention program to improve the national guidelines focusing on nutritional intervention and to improve patients' quality of life [13]. Therefore, this study aimed to assess nutritional recovery and its predictors among adult people living with HIV using a therapeutic feeding program in Finote-Selam General Hospital, Northwest Ethiopia.

## 2. Materials and Methods

### 2.1. Study Design and Setting

A retrospective cohort study was conducted in Finote-Selam Hospital, which is located in Northwest Ethiopia, and it is 375 km far from Addis Ababa, the capital city of Ethiopia, and 176 km away from Bahir Dar, the capital city of the Amhara regional state.

### 2.2. Populations

#### 2.2.1. Source Population

All recorded adult (≥18 years) HIV-infected people who were malnourished (MAM and SAM) and enrolled in the therapeutic feeding program between July 2010 and January 2017 in Finote-Selam Hospital.

#### 2.2.2. Study Population

All malnourished adult HIV patients who enrolled in a therapeutic feeding program between July 2010 and January 2017 in Finote-Selam Hospital and had recorded nutritional treatment outcomes are included.

### 2.3. Eligibility Criteria

#### 2.3.1. Inclusion Criteria

All malnourished adult HIV-infected people with documented nutritional treatment outcomes are included.

#### 2.3.2. Exclusion Criteria

Pregnant women and patients with body swelling (edematous patients) were excluded from the study because BMI measurement is not accurate for these groups of the population.

#### 2.3.3. Sample Size Determination and Sampling Procedure

The required sample size was calculated using Epi Info, version 7, statistical software with the following assumptions: 95% confidence interval, 80% power, and 1 : 1 exposed-to-nonexposed ratio. Sex was taken as an exposure variable because it yields the largest sample size and the percentage of recovery in the nonexposed group (female) is 67% from a recent study performed at Mekelle Hospital, Northern Ethiopia [14]. The final sample was considered 376.

Undernourished adult HIV-infected people who fulfill the inclusion criteria were selected from the available list of food by prescription registration booklet to develop a sampling frame and then the samples were selected using a simple random sampling method from available records.

### 2.4. Data Collection Procedure

Data were collected using a pretested structured checklist that was adapted from the WHO nutritional treatment guideline, Ethiopia Federal Ministry of Health food by prescription program registration book, patient chart, and follow-up form. The patient chart, food by prescription registration book, and HIV follow-up card were the source of data. The checklist includes sociodemographic data, clinical and laboratory data, ART-related data, and follow-up data. Data collectors (BSc Nurses) were trained on the objective of the study, the contents of the checklist, and how to extract data from different data sources.

### 2.5. Variables

The outcome variable was nutritional recovery (recovered/not recovered), which was defined as a participant reached a BMI of 18.5 or more for two consecutive visits within three months for moderate acute malnutrition and six months for severe acute malnutrition patients.

The independent variables were age, sex, religion, educational status, occupation, residence, ARV regimen, duration of ART, ART level of adherence, WHO clinical stage, opportunistic infection (OI), CD4 count, and functional status.

Moderate acute malnutrition is defined as BMI 16.5 kg/m^2^–18.5 kg/m^2^, and severe acute malnutrition is defined as BMI < 16.5 kg/m^2^. In this study, baseline clinical data means was at the time of malnutrition diagnosis. The level of ART adherence was classified based on the percentage of ARV drugs taken (Good ART adherence defined as 95% or greater of doses taken as prescribed, fair adherence defined as 85–94% of doses taken as prescribed, and poor adherence defined as less than 85% of doses taken as prescribed.) [15].

### 2.6. Data Analysis

Data were entered using Epi data, version 3.1, statistical software and analyzed using SPSS, version 20. Univariate analysis was used to describe the patient's baseline characteristics. The log-binomial regression model was used to identify predictors of nutritional recovery. In this study, we preferred log-binomial regression over the logistic regression model to report the adjusted risk ratio because the incidence of outcome (nutritional recovery) was greater than 10%.

## 3. Results

### 3.1. Sociodemographic Characteristics of Participants

In this study, the mean age of participants was 34 years (±9.2 SD) and the majority (60.2%) were females. Around 45.4% of the participants were married, and 62.0% were educated (Table 1).

From the total participants, 71.5% were treated as MAM cases and 78.7% were on ART at baseline. From patients on ART, about 42.3% had good ARV drug adherence. Nearly two-thirds of participants had WHO clinical stage I or II at baseline, and 54.0% had CD4 between 100 and 350 cells/*μ*L. The mean BMI was 16.86 (SD + 1.16) at baseline and 18.27 (SD + 1.29) at the end of the study (Table 2).

### 3.2. The Magnitude of Recovery from Malnutrition

In this study, after a median of five months of follow-up 14 weeks (IQR 10–19 weeks), 61.2% patients were considered recovered after they enrolled in the therapeutic feeding program. An overall total of 146 of 376 (38.8%) patients did not recover from undernutrition, 58 (15.4%) of whom died and 88 (23.4%) not responded. The nutritional recovery rate was 0.081 persons per month with a median recovery time of 12 weeks (IQR 9–17 weeks) for moderate acute malnutrition and 25 weeks (IQR 22–31 weeks) for severe acute malnutrition.

### 3.3. Factors Associated with Nutritional Recovery

In the current study, residence, educational status, ART adherence level, and WHO clinical stage were significant predictors of nutritional recovery at multivariable analysis.

It was found that participants with rural residences had a 47% lower probability of recovery when compared with urban (ARR = 0.53 (95% CI: 0.27–0.85)). Not attending any formal education was associated with a decreased probability of nutritional recovery (ARR = 0.24, 95% CI; 0.13–0.54). The probability of nutritional recovery was 86% lower for participants who had poor ART adherence compared with those who had a good adherence level (ARR = 0.14, 95% CI; 0.08–0.32). Similarly, the probability of nutritional recovery was 62% lower for participants who were WHO clinical stage III or IV at baseline as compared with those who were stage I or II (ARR = 0.38, 95% CI; 0.17–0.59) (Table 3).

## 4. Discussion

This study revealed that from the total patients who were on the therapeutic feeding program, nearly two-thirds of patients were recovered from undernutrition, which is comparable with a study done in Mekelle Hospital, Ethiopia [16], but it is higher when compared with studies conducted in different parts of sub-Saharan Africa including Ethiopia [17–20]. Most importantly, the current findings support WHO recommendations that nutritional counseling and therapy should be implemented for undernourished HIV patients. Furthermore, to improve treatment outcomes and quality of life, nutritional therapy and counseling interventions should be strengthened.

Our findings indicated that residence, educational status, ART adherence level, and WHO clinical stage were the predictors of nutritional recovery. A rural residence is described as a barrier to nutritional recovery. This was in concordance with other studies performed in different parts of Africa [17, 21]. The mechanism by which rural residence leads to poor nutritional recovery in HIV patients remains unclear, but it might be because rural settings would have high levels of poverty and difficulties in accessing different health services. Long-distance traveling to gather nutritional support every month might affect their adherence to the nutritional program. In line with the previous study [16], this study also revealed that those who did not attend any formal education decreased the probability of recovery from undernutrition. However, a study conducted at Gondar University Hospital in Ethiopia revealed that education is not significantly associated with nutritional recovery [20].

In the current study, the ART level of adherence was the other clinical predictor of nutritional recovery. The finding is in agreement with other studies [21, 22], suggesting that the poor level of ART adherence is associated with poor nutritional recovery. This could be described by the fact that poor ART adherence reduces immunity (CD4) and increases the occurrence of opportunistic infection. Food-based interventions can play a supportive role in overall weight gain and improving ART adherence. WHO clinical stage can predict many clinical outcomes in HIV patients. Similar to previous studies [16, 17, 19], the current study also showed that advanced WHO clinical stage (stage III or IV) decreases the probability of recovery from undernutrition.

The study has several limitations. First, the nature of the retrospective study design limits the ability to gather all relevant factors. The data were collected by documentary review, and hence, the analysis and interpretation of the data are restricted to only those variables that are captured in the patient records. Some of the important variables (socioeconomic status, food sharing at the household level, and distance to health institutions) were not accessible. Second, there could have been a selection bias arising from the fact that the samples were restricted only to patients with recorded nutritional outcomes. Lost to follow-up patients may have different characteristics than regular follow-up patients.

## 5. Conclusion

In this study, about 61.2% of the malnourished patients recovered after the therapeutic feeding program. This implies that nutrition plays a critical role in comprehensive care, support, and treatment of PLHIV. Therefore, incorporating nutrition interventions into HIV/AIDS programs is essential. Rural residence, no educational status, poor ART adherence level, and WHO clinical stage III or IV were the barriers for nutritional recovery for patients on the therapeutic feeding program. The finding of this study recommends that the therapeutic feeding program could improve the probabilities of nutritional recovery in malnourished HIV patients. In addition, the current finding suggests that a therapeutic feeding program should give more attention to persons with a poor adherence level, advanced WHO clinical stage, rural residence, and low educational status. Future research should be performed by including important variables such as socioeconomic status, food sharing at the household level, and distance to health institutions.

## Figures and Tables

**Table 1:** Sociodemographic characteristics of malnourished HIV patients, Finote-Selam Hospital, Northwest Ethiopia, January 2010–2017.

Variables	Category	Frequency (percentage)	
		Recovered	Not recovered
Sex	Male	90 (60.0%)	60 (40.0%)
	Female	140 (61.9%)	86 (38.1%)

Age	18–29	88 (38.3%)	48 (32.9)
	30–39	81 (35.2%)	61 (41.8)
	40–49	46 (20.0%)	27 (18.5)
	≥50	15 (6.5%)	10 (6.8)

Marital status	Not married	41 (17.8)	32 (21.9)
	Married	105 (45.7)	63 (43.2)
	Widowed	21 (9.1)	22 (15.1)
	Divorced	63 (27.4)	29 (19.8)

Occupation	Government employed	39 (17%)	21 (14.4)
	Unemployed	191 (83%)	125 (85.6)

Educational level	Not educated	89 (38.7%)	54 (37%)
	Educated	141 (61.3%)	92 (63%)

Residence	Urban	171 (74.3%)	104 (71.2%)
	Rural	59 (25.7%)	42 (28.8%)

**Table 2:** Baseline and follow-up clinical characteristics of adult malnourished HIV patients enrolled in the therapeutic feeding program, Finote-Selam Hospital, Northwest Ethiopia, 2010–2017.

Variables	Category	Frequency (percentage)	
		Recovered	Not recovered
ART status	On ART	190 (82.6%)	106 (72.6%)
	Pre-ART	40 (17.4%)	40 (27.4%)
Duration of ART	<6 month	65 (34.2%)	34 (32.0)
	6–12	14 (7.4%)	4 (3.8)
	>12 month	111 (58.4%)	68 (64.2%)
ART adherence level	Good	115 (61.2%)	44 (41.5)
	Fair/poor	73 (38.8%)	64 (58.8%)
WHO clinical stage	Stages I and II	152 (66.1%)	76 (52.1%)
	Stages III and IV	78 (33.9%)	70 (47.9%)
CD4 count	<100	57 (24.8%)	29 (19.9%)
	100–200	55 (23.9%)	47 (32.2%)
	200–350	63 (27.4%)	38 (26.0%)
	>350	55 (23.9%)	32 (21.9%)
Hemoglobin	<10	7 (8.6%)	12 (28.6%)
	10–11.99	10 (12.3%)	16 (38.1%)
	≥12	64 (79.0%)	14 (33.3%)
Functional status	Working	214 (93.0%)	117 (80.1%)
	Ambulatory/Bedridden	16 (7.0%)	29 (19.9%)
Baseline OIs	Yes	25 (10.9%)	35 (24.0%)
	No	205 (89.1%)	111 (76.0%)
Cotrimoxazole status	Yes	211 (91.7%)	128 (87.7%)
	No	19 (8.3%)	18 (12.3%)
INH status	Yes	57 (24.8%)	31 (21.2%)
	No	173 (75.2%)	115 (78.8%)
Other medication	Yes	16 (7.0%)	17 (11.6%)
	No	214 (93.0%)	129 (88.4%)
Type of malnutrition	MAM	186 (80.9%)	83 (56.8%)
	SAM	44 (19.1%)	63 (43.2%)

ART, antiretroviral treatment; INH, isoniazid; OI, opportunistic infection; WHO, World Health Organization.

**Table 3:** Multivariate analysis for the predictor of recovery from malnutrition, Finote-Selam Hospital, Northwest Ethiopia, 2010–2017.

Variables	Category	CRR (95% CI)	ARR (95% CI)
Sex	Male	1	1
	Female	1.09 (0.64–1.72)	1.61 (0.97–2.38)
Residence	Urban	1	1
	Rural	0.85 (0.32–0.93)^*∗*^	0.53 (0.27–0.85)^*∗*^

Educational status	Not educated	1.08 (0.76–1.32)	0.24 (0.13–0.54)^*∗∗*^
	Educated	1	1
Baseline CD4	<200	1	1
	201–500	1.25 (0.769–1.418)	1.23 (0.985–1.986)
	≥500	1.37 (1.115–1.680)^*∗*^	1.83 (0.83–2.21)
Adherence level	Good	1	1
	Fair or poor	0.44 (0.16–0.61)	0.14 (0.08–0.32)^*∗∗*^
WHO stage	Stage I or II	1	1
	Stage III or IV	0.72 (0.36–0.92)	0.38 (0.17–0.59)^*∗*^

^*∗*^ *p* value < 0.05; ^*∗∗*^*p* value <0.001. ARR, adjusted risk ratio; CRR, crude risk ration; WHO, World Health Organization.

## Data Availability

The data can be available from the corresponding author upon reasonable request.

## Abbreviations

AIDS: Acquired immunodeficiency syndrome
ARR: Adjusted risk ratio
ART: Antiretroviral therapy
BMI: Body mass index
CRR: Crude risk ratio
HIV: Human immunodeficiency virus
INH: Isoniazid
MAM: Moderate acute malnutrition
SAM: Severe acute malnutrition
WHO: World Health Organization.
